# Quantitative Analysis of the Impact of Finishing and Washing Processes on the Roughness of Polyester Fabric

**DOI:** 10.3390/polym16152199

**Published:** 2024-08-02

**Authors:** Ana Kalazić, Snježana Brnada, Tea Bušac, Tanja Pušić

**Affiliations:** 1 Department of Textile Design and Management, Faculty of Textile Technology, University of Zagreb, Prilaz baruna Filipovića 28a, 10000 Zagreb, Croatia; ana.kalazic@ttf.unizg.hr; 2 Department of Textile Chemistry and Ecology, Faculty of Textile Technology, University of Zagreb, Prilaz baruna Filipovića 28a, 10000 Zagreb, Croatia; tea.busac@ttf.unizg.hr (T.B.); tanja.pusic@ttf.unizg.hr (T.P.)

**Keywords:** roughness, woven fabric, fibers, polyester, chitosan, alkaline hydrolysis

## Abstract

The roughness of woven fabric surface has so far been mainly investigated as a key characteristic of comfort in contact with the skin. The analysis of roughness can be extended to various contexts and applications, becoming an important tool for understanding how textile materials react in interaction with different finishing agents, as well as for gaining insight into the durability and effectiveness of treatments. This research presents a comprehensive study on the impact of alkaline hydrolysis and chitosan coating on the roughness of polyester woven fabric, utilizing both novel and adapted methods. The study employed contact and optical methods to analyze fabric and fiber surface characteristics, highlighting the significance of roughness profile parameters in understanding material changes post-treatment. The investigation revealed that mechanical action, alkaline medium, washing temperature, and detergent residues contribute to fabric erosion and modification during washing, with chitosan coatings creating pronounced surface irregularities. Comparative analysis showed significant fabric roughness changes post-washing, while fiber roughness changes were treatment specific. Despite initial increases in fiber roughness due to treatments, their durability decreased after washing. These findings emphasize the importance of roughness analysis in optimizing textile finishing processes and washing cycles, impacting both comfort and treatment efficacy.

## 1. Introduction

Surface roughness plays a pivotal role in textile fabrics, influencing both consumer perception of garment quality and various production processes. It encompasses characteristics like unevenness, irregularity, and coarseness in texture, with its characterization considering wave amplitude and wavelength. The anisotropy of mechanical and geometrical properties, influenced by the arrangement and distribution of fibrous mass within a woven surface, further emphasizes its significance [1].

Surface roughness is a key factor in determining the tactile and functional properties of woven fabrics. Studies indicate that aspects like fabric structure, yarn density, and finishing processes can influence surface roughness, affecting the way of fabric feel and performance. However, traditional roughness measurement devices front challenges in capturing fine details due to the shape and size limitations of the stylus, pointing to the need for more accurate or alternative measurement techniques [2,3].

Many methods and techniques for determining the roughness of textile materials have been developed [4]. Initially, subjective assessments were prevalent, but since 1955, researchers have increasingly pursued quantitative methods to measure fabric surface characteristics. These methods fall into two broad categories: subjective and objective techniques. Objective techniques, which have gained prominence over time, encompass both contact and non-contact methods, each with its own set of advantages and limitations. Contact methods, while widely used, have notable limitations. They can damage the surface and provide erroneous results depending on the type and size of the contactor (stylus). Moreover, they are time-consuming. Additionally, contact methods are susceptible to environmental factors such as humidity, further complicating their reliability. 

A contact-type surface roughness measuring device, one of the most widely used methods, provides reliable measurements by directly contacting the sample [2]. However, such direct contact with the sample often carries a range of disadvantages, such as stylus wear, distortion of profile shape due to bending deformation, inability to measure viscous samples, limited measurement by the radius of the stylus tip, extended process, and difficulties in positioning and identifying subtle measurement points, as well as the need for sample preparation. On the other hand, advantages of this method include a clear wave profile and the ability to measure over greater distances.

As a response to these drawbacks, non-contact methods have gained popularity in recent years, despite the lack of standardization, leading to discrepancies in results [5,6]. The KES system (Kawabata Evaluation System) stands out as a commercially viable method, yet some studies suggest its results may be unrealistic due to its contact measurement mechanism [4]. Many researchers were dealing with the comparison of results from various instruments to identify the most accurate surface roughness measurement system, particularly tailored to textile surfaces. Furthermore, they emphasize the importance of comparing different surface roughness parameters to determine the most suitable ones for fabrics [7].

Contact methods involve direct contact between a sensor and the fabric surface during measurement. Various instruments have been developed for this purpose, including the Cloth Profile Recorder, KES, and the Fabric Touch Tester (Fabric Touch Evaluation System, FTT). These instruments measure surface roughness by quantifying the deviation in height along the fabric surface. Also, innovative optical methods for measuring fabric roughness through image processing can provide a more accessible approach compared to methods requiring expensive specialized equipment [8]. However, contact methods have the drawbacks mentioned above. However, the authors [4] state that, “The evaluation of roughness with hand consists of applying pressure on the fabric surface. Due to this fact, some researchers believe that the acquired results from contact methods are more compatible with subjective methods, and they could be the most appropriate techniques to measure surface roughness.”

The study has utilized objective methods to evaluate surface roughness and its correlation with various fabric properties. For instance, researchers have examined the effects of different woven structures and finishing processes on surface roughness [9]. Despite advancements in objective measurement techniques, challenges remain in standardizing methods and accurately interpreting results [2].

The basic factors contributing to the surface roughness of woven fabrics include yarn diameter, weave types, densities of weft, and warp, as well as the balance and cover factor of the fabric [10,11]. Additionally, studies have explored the relationship between surface roughness, friction, and tactile properties of fabrics. Studies showed that surface roughness is significantly affected by the type of fabric weave, with fabrics being rougher in the weft direction compared to the warp direction. It has been proven that initial surface roughness and fabric construction parameters significantly influence the changes caused by abrasion on fabric surfaces. Research findings indicate that as the number of abrasion cycles increases, the surface roughness of woven wool fabrics tends to decrease, mainly due to the reduction in irregularities caused by fiber ends protruding from the yarn [11].

The anisotropy in woven textile structures and complex weaving patterns contribute to variations in surface roughness, making it difficult to draw consistent conclusions. To address these issues, researchers have proposed novel approaches, including contactless image analysis and advanced spectral methods, to improve understanding of the impact of surface roughness on tactile perception and fabric quality [12].

**Figure 1:**
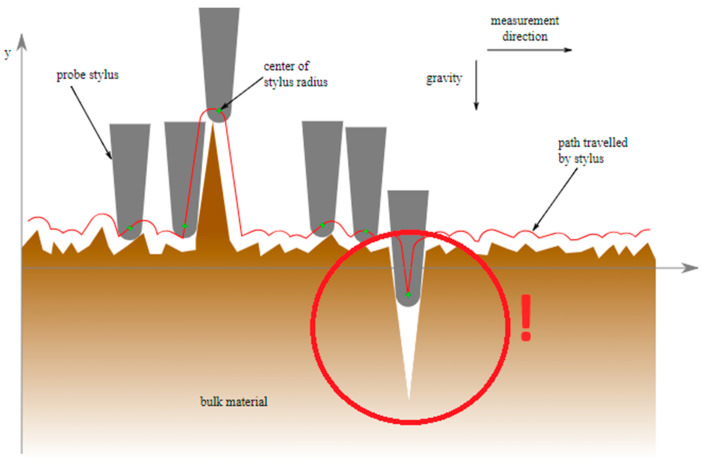
Stylus limitations in detecting deep valleys. Reproduced with permission from Emok, 2008. Permission is granted to copy, distribute, and/or modify this document under the terms of the GNU Free Documentation License, Version 1.2 or any later version published by the Free Software Foundation; with no Invariant Sections, no Front-Cover Texts, and no Back-Cover Texts. A copy of the license is included in the section entitled GNU Free Documentation License. (https://commons.wikimedia.org/wiki/Commons:GNU_Free_Documentation_License,_version_1.2 accessed on 15 May 2024).

Figure 1 shows how surface roughness measuring devices often have difficulty detecting narrow, deep valleys due to limitations related to the shape and size of the stylus. Because of the spherical shape of the stylus tip, if the width of the valleys is narrower than the radius of the stylus tip, the stylus will not be able to accurately follow the shape.

For this reason, characterization needs to be conducted at the yarn surface level. This involves a bundle of fibers that are aligned in parallel and then twisted torsionally. The fiber bundle contains pores and irregularities, with the outer layer of fibers defining the outer surface. Thus, finishing processes can affect the fabric surface. The roughness of the yarn surface, conditioned by its structure, will influence the capacity of the yarn surface, and consequently, the fabric, to adhere the finishing agent where applicable. This refers to the contact surface, the surface layer of the woven fabric. The larger the contact surface of the fabric, the greater the capacity to interact with the agent and the greater the effect of the agent on the contact surface (e.g., the skin).

Fabric roughness can also be linked to environmental impacts, particularly in the context of water repellency and other surface-oriented properties. The research revealed that the texture design of woven fabrics can play a key role in achieving higher water repellency, which could reduce reliance on environmentally harmful coatings [13,14]. Chitosan is a biopolymer used in numerous applications, including as a surface rheology modifier for polyester textiles to reduce the release of regular and/or irregular fragments during the washing process [15].

Polyester fibers have numerous advantages, which is confirmed by the increase in their usage compared to other synthetic fibers [16,17]. Their benefits include strength, durability, dimensional stability, resistance to chemicals, high resistance to microorganisms, UV radiation, and low cost. The disadvantages are static electricity buildup, low comfort, and recently, environmental concerns regarding the accumulation of microplastics. Some properties can be improved through various modifications, and one possibility is chitosan functionalization to reduce the release of fragments from polyester textiles during wash-ing [18]. 

In this study, the surface roughness of polyester woven fabric was analyzed before and after gradual modification, which included alkaline hydrolysis (surface scaling), chitosan coating, and the washing process—monitoring the stability of chitosan layered on the surface. Alkaline hydrolysis is a well-established method for modifying the surface of polyester fibers to enhance chitosan binding [19]. Using FTT, it was analyzed whether the modifications cause changes on the fabric surface, particularly in terms of fabric roughness at the fabric surface level (yarn + weave). To determine the effects of finishing processes and washing cycles on the topographical and morphological characteristics of the polyester fiber surface, an optical method was used to analyze surface roughness. Scanning electron microscopy (SEM) was employed to examine the morphological features of the fibers. The obtained results regarding changes in fiber surface characteristics are utilized to assess the impact of each finishing process and washing cycle on the fiber surface, where this level of surface roughness and morphology will not directly affect the sensory characteristics of the fabric surface.

## 2. Materials and Methods

For the purpose of this study, a standard polyester woven fabric (PN-01) from the supplier Center for Test Materials, CFT, Vlaardingen, The Netherlands, was selected. This fabric has a white tone and a surface mass of 156.0 g/m^2^. The thread density is 27.7 threads/cm in the warp and 20 threads/cm in the weft. The fabric thickness is 0.35 mm, while the yarn fineness is 30.4 tex for the warp and 31.9 tex for the weft. The fabric is woven in a plain weave, making it suitable for analysis in this study.

### 2.1. Fabric Samples for Testing—Sample Preparation

Considering that polyester as a synthetic polymer and chitosan as a biopolymer are incompatible, it was necessary to conduct a pretreatment through alkaline hydrolysis (AH). This treatment was carried out using a NaOH solution (w = 2%) at a temperature of 98 °C for 30 min in a laboratory device Polymat, Mathis, CHT Switzerland AG, Montlingen, Switzerland. After this thermochemical reaction, to remove oligomers from the surface of the polyester fabric, a gradual rinsing with water was conducted, first with hot water for two cycles and finally with cold water for two cycles. The samples were air-dried and stored in a closed space to avoid potential contamination from the environment.

**Figure 2:**
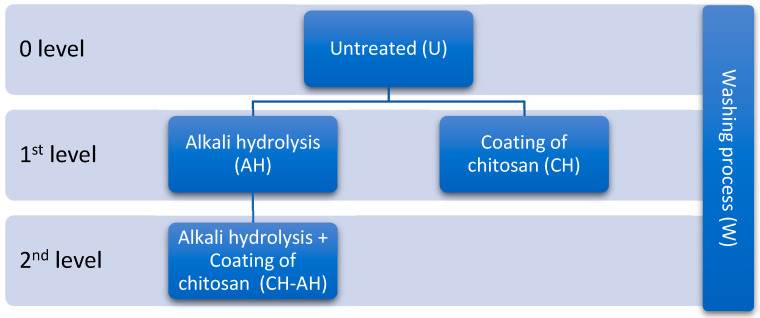
Sample Processing Levels.

As illustrated on Figure 2, a certain number of samples of standard polyester woven fabric, both before (U) and after alkaline hydrolysis (AH), were subjected to treatment with chitosan (CH) in concentration 0.5% prepared and stabilized with 1 mol/(L HCl. This treatment was performed with homogenous and pH-stabilized (pH 3.46) chitosan solution using an impregnation process on an Ernest Benz padder and stenter, LFV/2 350R + TKF 15/M350, with a pressure of 12.5 kg/cm. After impregnation, the fabric was dried in the modular unit at 90 °C for 40 s and cured at 130 °C for 20 s.

In the second stage, the stability of the chitosan coating on the surfaces of the initial and modified samples was analyzed during the washing process with reference ECE A detergent (1.25 g/L) at 60 °C with a bath ratio of 1:7 in a laboratory device Rotawash, SDL Atlas, Rock Hill, SC, USA. After each individual cycle, the samples were rinsed with water through four cycles with a bath ratio of 1:8. After completing 5 and 10 washing cycles, the samples were air-dried.

### 2.2. Surface Roughness Analysis of Fabric Samples Using FTT Device

Surface roughness analysis was conducted using the Fabric Touch Tester device, SDL ATLAS, Rock Hill, SC, USA. Since this paper is research oriented, sample testing was performed exclusively on the fabric front (both warp and weft directions), considering the method of modification, primarily involving chitosan coating.

**Figure 3:**
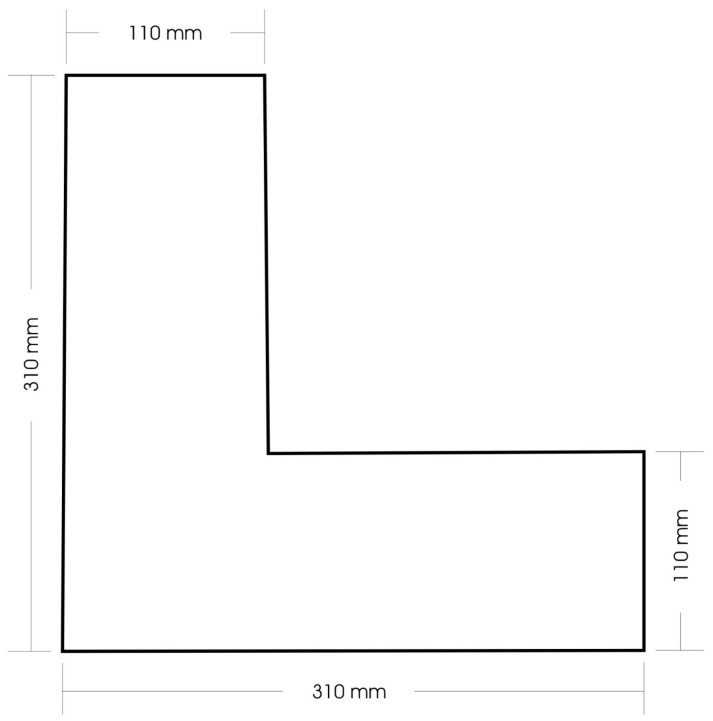
Fabric sample shape.

Prior to testing, the samples were prepared and conditioned in a standard atmosphere at 20 °C ± 2 °C with 65% ± 4% relative humidity. The sample was prepared using an ultrasonic cutter (Ultrasonic cutter for textiles TTS-400, Sonic, Rho, Italy) in the shape of the letter L with dimensions specified in the device’s operating instructions (see Figure 3).

During testing, the upper plate of the device is positioned on the sample, which begins to move vertically. The stylus starts measuring, providing insights into the surface roughness values simultaneously in both the warp and weft directions.

#### Methodological Issues and Adaptation of Data Analysis

Since the FTT provides only numerical assessment values and averages, additional data processing methods are required to gain deeper insights into the roughness of the tested fabric.

The software generates an index report consisting of 13 FTT index data indices. To obtain specific parameter data under observation, one needs to select the table. Given that this study focuses on surface roughness testing, the extracted data presented values of vertical and horizontal roughness, specifically warp and weft roughness, and distances that were equal in both directions. For profile processing, it is crucial that the distance between two points, or the observed period, remains consistent. The developed methodology allows for a detailed analysis of surface roughness at the yarn level, providing deeper insights into structural changes caused by coating of chitosan particles. Furthermore, it enables the assessment of the textile surface’s capacity to attract and mechanically bind certain finishing agents, crucial for the development of new textile products with enhanced properties.

### 2.3. Surface Roughness Analysis of Fibers Using MountainsLab Software

For the analysis of fiber roughness, a scanning electron microscope (FE-SEM), Mira II LMU (Tescan, Brno, Kohoutovice, Czech Republic), was used at a magnification of 1000×, and the images were processed using MountainsLab software, v. 10.1 to generate roughness profiles. Prior to analyzing the roughness profiles, it was necessary to set scales for each individual image to ensure the relevance of the roughness profile data. Measurements of roughness profiles were conducted on five specimens per SEM image to account for variability and ensure representativeness of the results.

For each identified fiber, the roughness profile was determined using the primary unfiltered profile, followed by an analysis of roughness based on the average value of the Rq parameter obtained from the five tested specimens. 

## 3. Results

The results of experimental section are divided into two parts. The first part involves the tactile examination of woven fabric samples at various stages of treatment and washing cycles using the contact method on the FTT device. The obtained results provided insights into changes in fabric surface characteristics due to exposure to different treatment processes and washing cycles, primarily at a macro level, including alterations in yarn structure and woven fabric structural properties (density, weave). Before proceeding with the testing, a methodological issue had to be addressed, which required modification in the approach to processing roughness profile data.

In the second part of the results, the roughness was also tested using an optical method at the fiber level, aimed at identifying changes in the polyester fiber itself resulting from the aforementioned processes. These results provided insights into surface changes of the fiber due to different treatments and washing processes, leading to conclusions about the effectiveness and durability of the treatments.

### 3.1. Surface Roughness Determination by Contact Method

Fabric samples were tested using the contact method on the Fabric Touch Tester (FTT) device on the front side, simultaneously in both the warp and weft directions (vertical and horizontal). The sample is placed on the testing device and probed by a stylus. When the plate applies pressure to the sample, it begins to move, and the stylus, under the applied pressure force, starts measuring, providing insights into the surface roughness values.

#### 3.1.1. Methodological Issues—Geometry and Dimensions of the Stylus

The issue with stylus geometry and dimensions arises when the stylus tip is relatively larger compared to the relief elements of the fabric. Considering that the stylus is a cylindrical rod with a semi-spherical head (diameter 1 mm), adjustments were made to the method for determining the surface roughness parameters of the fabric structure segments.

**Figure 4:**
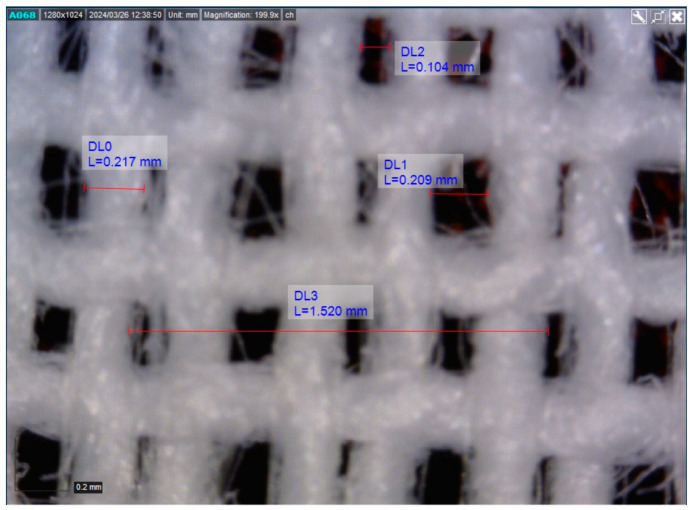
Dino-Lite image of tested woven fabric.

Figure 4 show microscopic images obtained using the Dino-Lite device reveal irregularities in the warp density—two adjacent warp yarns are closer together due to 2/2 reed threading. These observed irregularities in warp density can cause local variations in the fabric structure, making it crucial to analyze the fabric surface based on structural segments and their dimensions to understand how these irregularities affect the roughness profile.

- Thickness of the marked yarn: DL0 = 0.217 mm
- Width of the marked larger pore: DL1 = 0.209 mm
- Width of the marked smaller pore: DL2 = 0.104 mm
- Vertical contact width of the path: D03 = 1.520 mm
- Maximum profile depth (when the stylus probes a vertical pore, theoretically equal to the fabric thickness of 0.35 mm) due to dimensional characteristics of the fabric surface relief and stylus—could not be measured.

**Figure 5:**
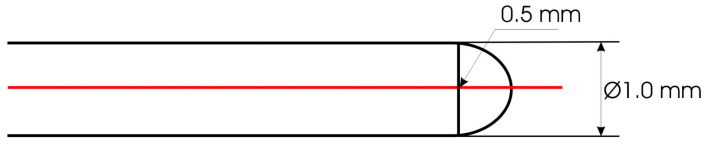
Side view of a hemispherical stylus.

In Figure 5, a side view of a hemispherical stylus is shown. The semi-spherical stylus moves across the surface, probing the profile with its spherical cap. Given the contact width of the path of 1.520 mm, 0.76 mm on either side of the central point (D03), any contact with relief outside the central part of the stylus can cause lateral shift that will not be visible in the roughness profile (Equation (1)). This situation can lead to misinterpretation of the profiles, as it may actually measure two or more roughness profiles with lateral displacement, resulting in profiles with higher peaks.

(1) $$a=1\;mm-0.35\;mm=0.65\;mm\;b^{2}=1\;mm-0.4225\;mm\;b=0.76\;mm$$

**Figure 6:**
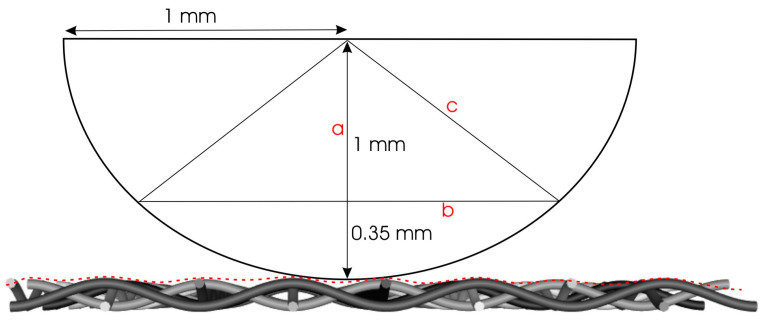
Technical scheme of semi-spherical stylus tip with illustration of the obtained and actual roughness profiles resulting from the stylus geometry and dimensions.

This disproportion presents a challenge when attempting to define surface roughness per weave unit, as the semi-spherical shape of the stylus obstructs precise detection. Although the stylus detects the profile at the yarn level, it must be considered that the results are not the outcome of simple linear movement of the stylus across the surface. Instead, the stylus captures only the highest points of relief during its movement, leading to uncertainty in interpreting the results. Thus, the obtained overall profile is actually a compilation of random segments of the yarn surface rather than a linear continuous profile (Figure 6). 

Therefore, these results have to be considered as a unified linear profile, even though it actually represents segments of the fabric surface captured during the stylus movement. After defining the domain, specifically the boundary values of the range, the data have to be filtered and analyzed using statistical software.

#### 3.1.2. Modification of Processing Method and Data Analysis

During FTT data analysis to determine roughness parameters, the device software considers data from the primary profile, which includes multiple levels of roughness. This includes waviness caused by global irregularities in the fabric (such as weave structures, folds, etc.) and roughness at the yarn surface level, which is influenced by the parallelized, torsionally twisted fibers. The roughness results generated in the FTT report actually reflect the waviness of the profile and do not provide insights into the surface roughness at the yarn level. Therefore, prior to processing the profile, it is necessary to filter it by extracting all large-scale lengths, as illustrated in the figure from the primary profile.

**Figure 7:**
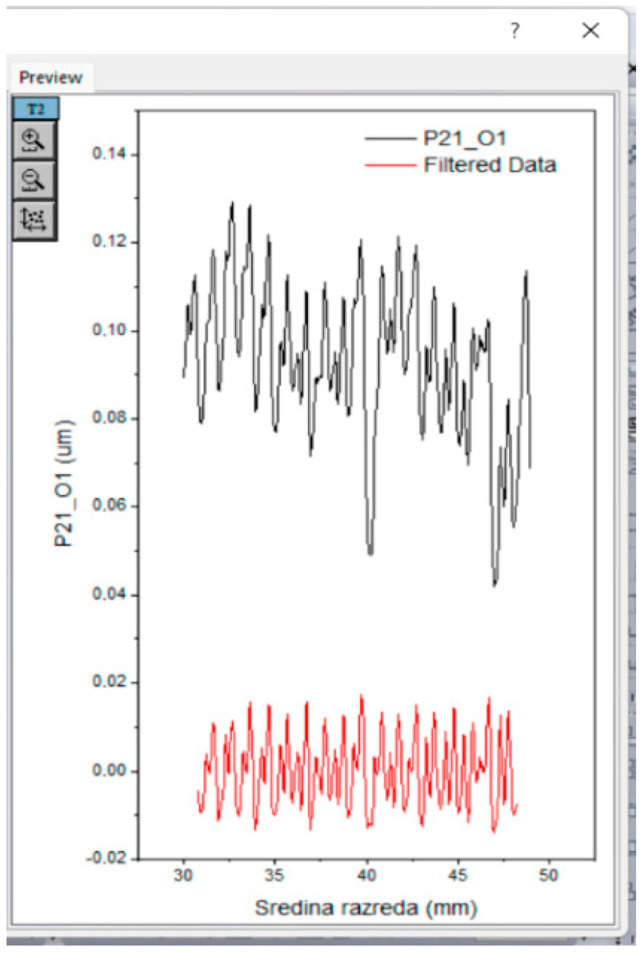
Example of data filtering—from original profile to filtered profile.

For the purpose of analysis, the data has been grouped so that the distance period for all points in the profile is equal. The roughness profile has been filtered to exclude wavelength components greater than 0.5 mm (cutt of) to avoid weave-related roughness and reduce the influence of the semi-spherical shape of the stylus, thereby achieving higher precision (Figure 7). Extremes that do not belong to the natural surface topography of the fabric have been identified, and valid ranges of data have been determined for processing, ensuring that the length of the profile evaluated is not less than five cutoff lengths.

This approach enables a better understanding of the yarn’s relief structure and potential errors due to mismatched stylus geometry. 

From the primary profile, all wavelength components that are equal to or greater than 0.5 mm have been excluded, thus eliminating the influence of the weave (warp and weft densities) on surface roughness. Three specimens were measured for each sample and combined into one profile. During data processing, three identical profiles were measured, and basic statistical indicators were calculated. However, the roughness parameter itself is a statistical indicator equivalent to standard deviation or the square deviation of the roughness profile.

The warp density of the fabric sample is more than 30% higher than the weft density, while the differences in their fineness are insignificant. During the washing process, typically in a washing machine under standard conditions, the weft yarns have greater freedom of lateral movement compared to the warp yarns, which are tightly packed in the structure. Due to the hydrodynamic action and rotation of the drum during washing, the fabric bends, experiencing abrasion, and partially releases chitosan, or sheds fragments from its surface due to the alkalinity of the wash bath, temperature, and other factors defined by Dr. Herbert Sinner in 1964. These results show that during the washing process, despite random stresses, the fabric does not behave uniformly in all directions but depends on its potential for interstructural movement in specific directions. 

**Figure 8:**
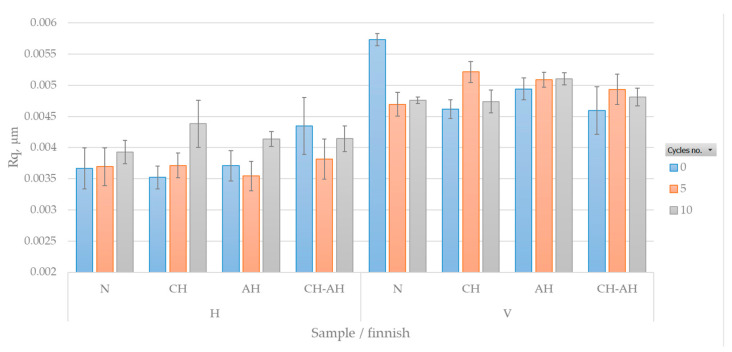
Average squared deviation of the fabric surface roughness profile.

In Figure 8, the results of the average squared deviation of the surface roughness profile heights filtered at a cutoff of 0.5 mm short wavelength are graphically displayed, in sequence—from the fabric front.

The chart is divided into two parts—H (horizontal roughness, i.e., roughness in the weft direction) and V (vertical roughness, i.e., roughness in the warp direction). The tested samples are as follows: U—untreated, control samples; CH—samples treated with chitosan; AH—alkaline hydrolyzed samples; CH-AH—alkaline hydrolyzed samples treated with chitosan. The color of the bars represents the number of washing cycles (5 and 10) to which each sample was subjected (graph legend). From the results shown in Figure 8, it is evident that there is no significant difference in the Rq parameter values based on the type and degree of sample treatment. Roughness increases with washing cycles, which is more pronounced after 10 washing cycles, while after 5 washing cycles, roughness can temporarily decrease (especially in alkaline hydrolyzed samples).

The untreated sample (U) before and after 5 and 10 washing cycles in the weft direction exhibits lower roughness, while it shows the opposite trend in the warp direction. With washing cycles, a slight increase in roughness is observed in the weft direction. The untreated sample shows the highest roughness, particularly in the warp direction without washing cycles. In contrast to the weft direction, roughness decreases with increasing washing cycles in the warp direction. The results indicate that washing cycles impact changes in the fabric surface topography in the main axis. The cause could be changes in the fabric structure due to mechanical action, alkaline environment, detergent composition, hard water, and washing temperature, as well as residues from detergent and calcites originating from hard water.

**Figure 9:**
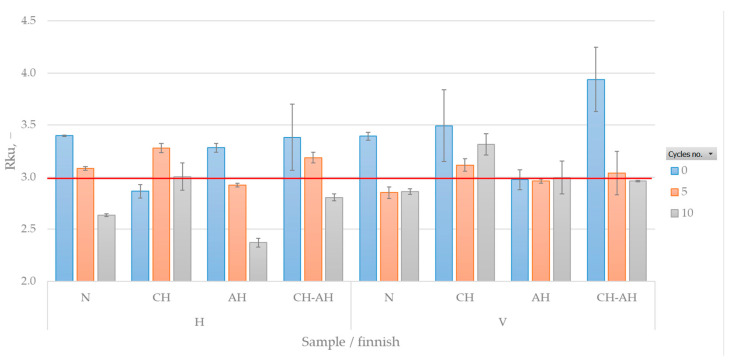
Kurtosis of the roughness profile.

In Figure 9 it could be seen that the roughness profile increases with washing cycles (Rku takes lower values). Alkaline hydrolysis affects the surface morphology by resulting in smoother peaks. Alkaline hydrolysis causes the surface to peel, resulting in a loss of mass and decreased tensile properties, and creates a new, more porous and airy structure.

Through uneven deposition of chitosan on the peeled alkaline hydrolyzed surface of the fabric, there were localized areas with higher accumulations of chitosan, leading to more pronounced peaks and valleys on the fabric surface.

**Figure 10:**
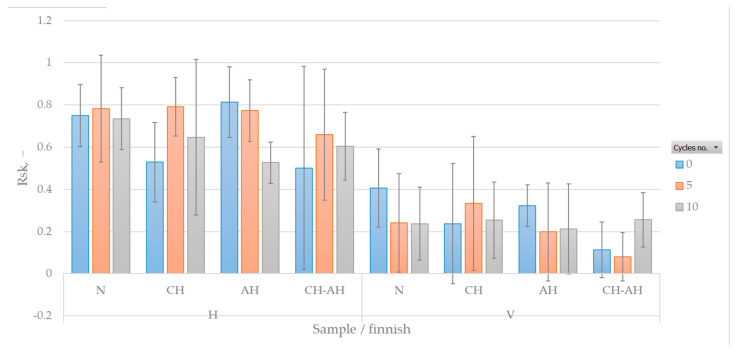
Skewness of the roughness profile.

Figure 10 shows the measure of asymmetry (skewness) of the surface, specifically the degree of bias in the roughness profile (asperity shape).

From the results, it can be concluded that the distribution of peaks and valleys in the fabric is influenced by the density of the yarns, where the warp density is higher than the weft density, while the fineness of the yarns used for warp and weft is approximately equal. During vertical probing using the FTT (warp direction), where the weft is probed transversely, peaks are less pronounced due to the higher density of warp yarns, resulting in a distribution of peaks and valleys closer to a symmetric distribution. On the other hand, probing the weft (horizontal) shows more pronounced peaks due to the lower density of the weft, leading to greater asymmetry in the surface, specifically in the distribution of peaks and valleys.

### 3.2. The Optical Method for Measuring Fiber Surface Roughness

**Figure 11:**
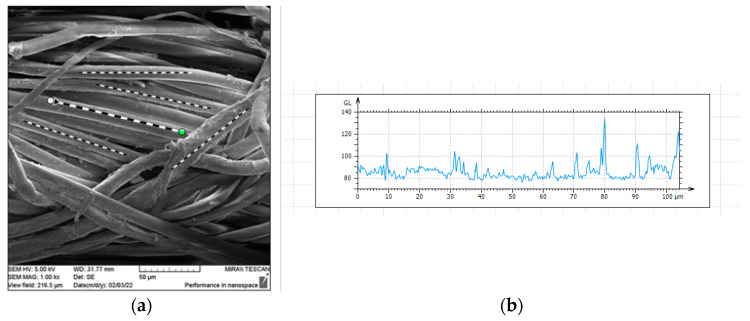
Analysis in MountainsLab software: (**a**) Measurement of roughness profiles on five specimens (Illustration); (**b**) Primary unfiltered profile graph (Illustration).

With the help of MountainsLab software, optical analysis of fiber roughness was conducted, with the roughness of five samples measured on each SEM image (Figure 11a). Images were obtained using a scanning electron microscope (SEM) and subsequently processed to obtain the roughness profile for each tested fiber (Figure 11b). 

**Figure 12:**
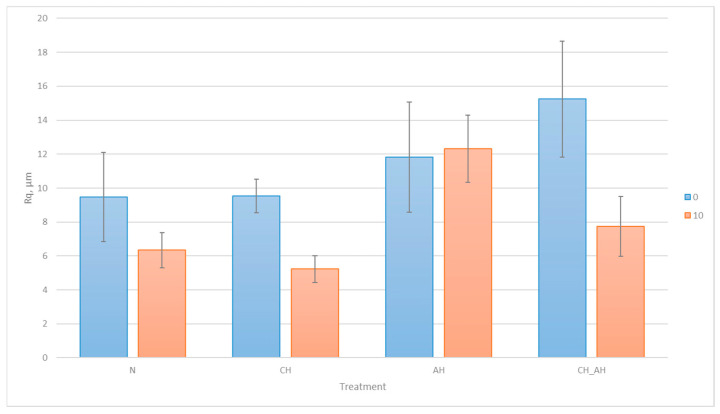
Average squared deviation of the fiber surface roughness profile.

The average roughness of each tested fiber, before and after 10 washing cycles, is shown in Figure 12.

In Figure 12, the results of the average squared deviation of the fiber surface roughness profile heights are graphically displayed for each fiber sample, before and after 10 washing cycles.

During the analysis of roughness of differently treated polyester fibers before the washing process, it was observed that there is no significant difference in the longitudinal roughness between untreated polyester fiber and polyester fiber treated with chitosan. Alkaline hydrolyzed polyester fiber exhibits higher roughness compared to untreated polyester fiber and polyester fiber treated with chitosan. However, the longitudinal roughness is significantly higher in polyester fiber that was first alkaline hydrolyzed and then treated with chitosan.

Chitosan is applied to the fiber in the form of a gel, covering the entire surface of the fiber while retaining its initial roughness. Since chitosan particles are positively charged and repel one another, the uniform coverage does not contribute significantly to changes in longitudinal roughness, unless there are additional surface irregularities that the gel could fill or emphasize.

Alkaline hydrolysis of polyester fiber causes surface scaling and predominantly longitudinal crater formation, resulting in increased surface roughness of the fiber. The significantly higher roughness observed in the sample first alkaline hydrolyzed and then treated with chitosan can be attributed to the combined effect of surface scaling caused by alkaline hydrolysis and subsequent chitosan treatment, which further emphasizes the existing surface irregularities.

After 10 washing cycles, a noticeable decrease in roughness is evident in all observed fiber samples, except for the alkaline hydrolyzed sample. In the untreated sample, the reduction in roughness could be attributed to the filling of any irregularities in the roughness profile with detergent residues. The roughness of chitosan-treated fibers further decreased, indicating potential stabilization during the washing process, where residues from hard water may fill remaining voids. There was a slightly significant increase in roughness observed in the alkaline hydrolyzed fiber after 10 washing cycles. This could be due to the already compromised surface of the fiber further eroding due to Sinner’s circle, resulting in increased roughness.

Although the initial treatments with alkaline hydrolysis followed by chitosan increased the surface roughness of the fibers, the stability of their binding was not permanent after exposure to washing cycles, as evidenced by a drastic reduction in the roughness profile.

## 4. Conclusions

The study of fabric surface roughness in this research revealed significant changes caused by different treatment levels and washing cycles. When the FTT stylus device probes the surface vertically (along the fabric’s warp direction), it measures the roughness of the weft threads. Results show that roughness decreases with increasing washing cycles in a vertical direction, possibly due to fabric shrinkage and mechanical action smoothing the fabric surface. In contrast, in the horizontal direction (along the weft direction), roughness becomes more pronounced due to lower thread density, resulting in greater asymmetry.

The distribution of peaks and valleys in the fabric depends on the thread density at the macro level. During vertical probing (warp direction), peaks are less pronounced due to higher warp thread density, resulting in a more even surface. During horizontal probing (weft direction), peaks are more pronounced due to lower weft density, leading to greater surface asymmetry.

These results highlight how roughness profile parameters can be used to gain deeper insights into changes in textile materials after specific treatments. Changes in fabric structure after washing may be caused by a combination of mechanical action, alkaline medium, washing temperature, and residues from detergent and hard water, resulting in fabric erosion and modification. Uneven chitosan coating on alkaline hydrolyzed fabric creates localized accumulations, causing more pronounced peaks and valleys on the fabric surface.

Analysis of differently treated polyester fibers before washing showed no significant difference in longitudinal roughness between untreated and chitosan-treated polyester fibers. However, alkaline hydrolyzed fibers exhibited greater roughness due to surface erosion caused by alkaline hydrolysis. After 10 washing cycles, roughness further increased due to continued erosion of the already weakened surface. These changes in micro-level roughness provide insights into the durability and efficiency of treatments.

Comparison of fabric and fiber roughness indicated that fabric surface roughness changes significantly after washing, while changes in fiber roughness are specific to the type/degree of treatment. Although treatments with alkaline hydrolysis and chitosan initially increased fiber roughness, their binding durability was not permanent after exposure to washing cycles, resulting in reduced roughness profiles. These findings underscore the importance of roughness profile parameters for understanding and optimizing finishing processes and washing cycles of textile materials.

The ability to analyze fabric and fiber roughness provides valuable insights into changes that impact not only comfort but also the durability and efficiency of treatments. Future research should consider the anisotropy of fabric surface shape in the washing process when observing roughness changes due to further treatments of samples.

## Data Availability

The original contributions presented in the study are included in the article; further inquiries can be directed to the corresponding authors.

## References

[1] Militky J., Nováková J. (2004). Anisotropy of Fabric Surface Roughness. Proceedings of the Magic World of Textiles.

[2] Palová K., Kelemenová T., Kelemen M. (2023). Measuring Procedures for Evaluating the Surface Roughness of Machined Parts. Appl. Sci..

[3] Kassaw A., Ayele M. (2024). Effect of Weft Yarn Type and Weaving Parameters on Surface Roughness and Drapeability of Woven Fabric. J. Nat. Fibers.

[4] Mooneghi S.A., Saharkhiz S., Varkiani S.M.H. (2014). Surface Roughness Evaluation of Textile Fabrics: A Literature Review. J. Eng. Fibers Fabr..

[5] Aulbach L., Salazar Bloise F., Lu M., Koch A. (2017). Non-Contact Surface Roughness Measurement by Implementation of a Spatial Light Modulator. Sensors.

[6] Lishchenko N., O’Donnell G.E., Culleton M. (2023). Contactless Method for Measurement of Surface Roughness Based on a Chromatic Confocal Sensor. Machines.

[7] Wagner M., Michaud M. A Comparison of Surface Roughness Measurement Methods for Gear Tooth Working Surfaces. Proceedings of the AGMA American Gear Manufacturers Association 2019 Fall Technical Meeting.

[8] Beyene K.A., Gebeyehu E.K., Adamu B.F. (2023). The Effects of Pretreatment on the Surface Roughness of Plain-Woven Fabric by the Kawabata Evaluation System. Text. Res. J..

[9] Vassiliadis S., Matsouka D., Watel Q., Jaouani H. (2020). Optical Method for the Determination of the Roughness Profile of Woven Fabrics. IOP Conf. Ser. Mater. Sci. Eng..

[10] Akgun M. (2016). Surface Roughness Properties of Wool Woven Fabrics after Abrasion. J. Text. Inst..

[11] Militk J. (2012). Woven Fabrics Surface Quantification. Woven Fabrics.

[12] Jonas A.M., Cai R., Vermeyen R., Nysten B., Vanneste M., De Smet D., Glinel K. (2020). How Roughness Controls the Water Repellency of Woven Fabrics. Mater. Des..

[13] Pellis A., Guebitz G.M., Nyanhongo G.S. (2022). Chitosan: Sources, Processing and Modification Techniques. Gels.

[14] Morin-Crini N., Lichtfouse E., Torri G., Crini G. (2019). Applications of Chitosan in Food, Pharmaceuticals, Medicine, Cosmetics, Agriculture, Textiles, Pulp and Paper, Biotechnology, and Environmental Chemistry. Environ. Chem. Lett..

[15] Kang H., Park S., Lee B., Ahn J., Kim S. (2021). Impact of Chitosan Pretreatment to Reduce Microfibers Released from Synthetic Garments during Laundering. Water.

[16] Rajak D.K., Wagh P.H., Linul E. (2022). A Review on Synthetic Fibers for Polymer Matrix Composites: Performance, Failure Modes and Applications. Materials.

[17] Gonzalez V., Lou X., Chi T. (2023). Evaluating Environmental Impact of Natural and Synthetic Fibers: A Life Cycle Assessment Approach. Sustainability.

[18] Pušić T., Kaurin T., Liplin M., Budimir A., Čurlin M., Grgić K., Sutlović A., Volmajer Valh J. (2023). The Stability of the Chitosan Coating on Polyester Fabric in the Washing Process. Tekstilec.

[19] Kaurin T., Pušić T., Čurlin M. (2022). Biopolymer Textile Structure of Chitosan with Polyester. Polymers.

